# The effect of age on the clinical and immune characteristics of critically ill patients with COVID-19: A preliminary report

**DOI:** 10.1371/journal.pone.0248675

**Published:** 2021-03-18

**Authors:** Chunling Hu, Junlu Li, Xia Xing, Jing Gao, Shilong Zhao, Lihua Xing

**Affiliations:** Department of Respiratory Intensive Care Unit, The First Affiliated Hospital of Zhengzhou University, Zhengzhou, Henan Province, China; University of Sassari, ITALY

## Abstract

**Background:**

In December 2019, a new disease named coronavirus disease 2019 (COVID-19) was occurred. Patients who are critically ill with COVID-19 are more likely to die, especially elderly patients. We aimed to describe the effect of age on the clinical and immune characteristics of critically ill patients with COVID-19.

**Methods:**

We retrospectively included 32 patients with COVID-19 who were confirmed to have COVID-19 by the local health authority and who were admitted to the first affiliated hospital of Zhengzhou University in Zhengzhou, China between January 3 and March 20, 2020. Clinical information and experimental test data were retrospectively collected for the patients. The 32 patients in this study were all in a critical condition and were classified as severe, according to the guidelines of 2019-nCoV infection from the National Health Commission of the People’s Republic of China. Data were compared between those <60 years old and ≥60 years old.

**Results:**

Of 32 patients, 13 were under 60 years old, and 19 patients were ≥60 years old. The most common symptom among all patients upon admission was fever (93.8%, 30/32). Compared to younger patients, older patients exhibited increased comorbidities. Among patients who were 60 years and older, platelet count, direct bilirubin (DBIL), indirect bilirubin(IBIL), lactate dehydrogenase (LDH), B-type natriuretic peptide (BNP), C-reactive protein (CRP), procalcitonin (PCT), and interleukin-10 (IL-10) were significantly higher than in younger patients who were less than 60 years old. CD4+ T lymphocytes, CD8+ T lymphocytes, and NKT lymphocytes were decreased, CD4+/CD8+ T lymphocytes were significantly increased in all 32 patients, while there were no evident differences between younger and older patients. The CURB-65 (confusion, urea, respiratory, rate, blood pressure plus age ≥65 years), Acute Physiology and Chronic Health Evaluation (APACHE) II and pH value were significantly higher in older patients than in patients who were under 60 years old. However, the PaO2 and PaO2:FiO2 were lower in older patients than the younger. Compared to patients under 60 years old, patients who were 60 years and older tended to develop ARDS (15 [78.9%] vs 5 [38.5%]), septic shock (7 [36.8%] vs 0 [0.0%]) and were more likely to receive mechanical ventilation (13 [68.4%] vs 3[23.1%]). Dynamic trajectories of seven laboratory parameters were tracked on days 1, 3, 5 and 7, and significant differences in lymphocyte count (*P* = 0.026), D-dimer (*P* = 0.010), lactate dehydrogenase (*P* = 0.000) and C-reactive protein (*P* = 0.000) were observed between the two age groups.

**Conclusions:**

A high proportion of critically ill patients were 60 or older. Furthermore, rapid disease progression was noted in elderly patients. Therefore, close monitoring and timely treatment should be performed in elderly COVID-19 patients.

## Introduction

In December 2019, a novel severe acute respiratory syndrome coronavirus 2 (SARS-CoV-2) was reported in Wuhan and quickly spread in and beyond China [1]. As of February 27, 2020, there have been more than 70,000 diagnosed cases and 2,747 confirmed deaths in China. SARS-CoV-2 belongs to the lineage of genus beta-coronavirus of the Coronavirus family, which includes SARS-CoV and MERS-CoV [2]. Similar to SARS in 2003, COVID-2019 conveys a high possibility of admission to the intensive care unit (ICU) and mortality [3]. Notably, older critically ill COVID-19 patients are at an increased risk for death [4–7], while few studies have compared differences in clinical features and laboratory finding in patients of different ages.

In this study, we retrospectively reviewed clinical data of critically ill patients with COVID-19 who were admitted to the First Affiliated Hospital of Zhengzhou University in Zhengzhou, Henan and determined differences in critically ill patients with respect to clinical features and laboratory findings in different age groups. Our study might provide new insight into the risk stratification and targeted therapeutic strategies for elderly COVID-19 patients.

## Methods

### Patient population

Thirty-two patients with COVID-19 were admitted to the First Affiliated Hospital of Zhengzhou University in Zhengzhou, China between January 3 and March 20, 2020. The all patients were confirmed via real-time reverse transcriptase polymerase chain reaction (RT-PCR) of throat-swab specimens from the upper respiratory tract or sputum specimens from the lower respiratory tract. The elderly patients (≥60 years old) tended to have more severe illness conditions. The reports to date, indicated that elderly patients of COVID-19 were likely to have more serious illness, a selective analysis of clinical and immune characteristics of COVID-19 in different ages remains to be performed. The nonelderly group (age <60 years old) and the elderly group (age≥60 years old) were divided by age. A diagnosis of pneumonia of unknown cause was performed according to the World Health Organization interim guidance. The epidemiological history, the viral titers, clinical characteristics, chest imaging, and exclusion of common pathogens are all diagnostic criteria for COVID-19. The 32 patients in this study were all in critical condition and were classified as severe, according to guidelines of the 2019-nCoV infection from the National Health Commission of the People’s Republic of China. All data were anonymous, and the requirement for informed consent was waived. Three medical doctors in the respiratory department at the First Affiliated Hospital of Zhengzhou University reviewed the medical records of all patients. This study was performed in accordance with guidelines and was approved by the Ethics Committees from the First Affiliated Hospital of Zhengzhou University.

### Data collection

Clinical information, including age, gender, APACHE II score, risk factors for critically ill patients, medical history, ventilation status, epidemiological history, demographic data, symptoms, underlying comorbidities, clinical charts, nursing records, laboratory findings, and chest X-rays, were retrospectively collected for all patients with COVID-19 as confirmed by the local health authority. All data were retrospectively obtained from the electronic medical records in the First Affiliated Hospital of Zhengzhou University. Three medical doctors in the First Affiliated Hospital of Zhengzhou University independently reviewed the data for all patients. Past medical history included surgical history, smoking history, and medical history, such as occurrence of malignant tumors, liver cirrhosis, hypertension and diabetes. Experimental examinations were as follows: PaO_2_, CD4^+^T lymphocyte cell count, CD8^+^T lymphocyte cell count, B lymphocyte cell count, levels of interleukin-6 (IL-6), IL-10, Interferon-r (IFN-r), tumor necrosis factor-a (TNF-a), procalcitonin (PCT), C-reactive protein (CRP), aspartate aminotransferase (AST), alanine aminotransferase (ALT), De Ritis Ratio, (AST/ALT), alkaline phosphatase (ALP), total bilirubin (TBIL), direct bilirubin (DBIL), indirect bilirubin (IBIL), lactate dehydrogenase (LDH), troponin (Tn), and brain natriuretic peptide (BNP). All blood samples were transferred to the designated laboratory in the First Affiliated Hospital of Zhengzhou University.

### Statistical analysis

Normally distributed continuous variables are presented as the mean ± SD and were compared by Student’s t-test, and nonnormally distributed continuous variables are shown as median with interquartile range (IQR) and were analyzed by Mann–Whitney test or Kruskal–Wallis test. Categorical variables are presented as percentages and were analyzed by Chi-square test or Fisher’s exact test. Generalized linear mixed models examined differences in laboratory data between patients <60 years old and ≥60 years old over time. All data were tested using SPSS software version 26.0 (SPSS, Chicago, IL, USA) and GraphPad Prism 8 (GraphPad Software Inc., La Jolla, CA, USA). Tests were two-sided, and p-values less than 0.05 were considered statistically significant.

## Results

### Baseline clinical features

Thirty-two critically ill patients were included in this study. Among these patients, 13 were under 60 years old, and 19 were greater than or equal to 60 years old. Baseline clinical features of severe and critically ill COVID-19 patients between the nonelderly (age < 60 years old) and elderly (age ≥60 years old) groups are shown in **Table 1**. 53.1% of critically ill patients were male, while 68.4% of male patients were 60 years and older. The most common presentation symptom was fever (93.8%, 30/32), followed by cough (43.8%, 14/32), fatigue (34.4%, 11/32), expectoration (18.8%, 6/32), headache (12.5%, 4/32), and diarrhea (9.4%, 3/32). Many patients had underlying comorbidities, including COPD (12.5%, 4/32), hypertension (31.3%, 10/32), diabetes (25.0%, 8/32), cardiovascular disease (12.5%, 4/32), cerebrovascular disease (12.5%, 4/32), chronic kidney disease (9.4%, 3/32) and two or more comorbidities (46.9%, 15/32). The mortality of patients was 21.9%, and dead patients were significantly older. The median time from onset to diagnosis, ICU, and mechanical ventilation was 7 days (IQR, 5–11), 18.0 days (IQR,12.25–21.75), and 14 days (IQR,9–19), respectively.

**Table 1:** Baseline clinical features of subjects with COVID-19 in different ages.

	All patients(n = 32)	Age <60 years old(n = 13)	Age≥60 years old(n = 19)	*P*- value
**Sex**–No., %				
male	17(53.1%)	4(30.8%)	13(68.4%)	0.070
**Exposure**–No., %	25(78.1%)	10(76.9%)	15(78.9%)	1.000
**Entering Symptoms**–No., %				
Fever	30(93.8%)	12(92.3%)	18(94.7%)	1.000
Cough	14(43.8%)	5(38.5%)	9(47.4%)	0.725
Expectoration	6(18.8%)	1(7.7%)	5(26.3%)	0.361
Fatigue	11(34.4%)	3(23.1%)	8(42.1%)	0.450
Headache	4(12.5%)	3(23.1%)	1(5.3%)	0.279
Diarrhea	3(9.4%)	2(15.4%)	1(5.3%)	0.552
**Comorbidities**–No., %				
≥two comorbidities	15(46.9%)	5(38.5%)	10(52.6%)	0.491
COPD	4(12.5%)	0(0.0%)	4(21.1%)	0.128
Hypertension	10(31.3%)	4(30.8%)	6(31.6%)	1.000
Diabetes	8(25.0%)	3(23.1%)	5(26.3%)	1.000
Cardiovascular disease	4(12.5%)	0(0.0%)	4(21.1%)	0.128
Cerebrovascular disease	4(12.5%)	1(7.7%)	3(15.8%)	0.629
Chronic kidney disease	3(9.4%)	2(15.4%)	1(5.3%)	0.552
**Time from onset to, media(IQR), d**				
diagnosis	7(5–11)	9(3–13.5)	7(5–9)	0.448
ICU	18.0(12.25–21.75)	20.0(12.5–25.5)	17(12–19)	0.170
mechanical ventilation	14(9–19)	14(10.5–19.5)	11(8.5–19)	0.467

**Abbreviations:** ARDS, Acute respiratory distress syndrome; HBV, Hepatitis B virus; COPD, Chronic obstructive pulmonary disease.

Compared to younger COVID-19 patients under 60 years old, comorbidities, including COPD (21.1% vs 0.0%), hypertension (31.6% vs 30.8%), diabetes (26.3% vs 23.1%), cardiovascular disease (21.1% vs 0.0%), and cerebrovascular disease (15.8% vs 7.7%) were more prevalent in the elderly group, while chronic kidney disease was higher in the nonelderly group (15.4% vs 5.3%). In addition, the median time from onset to diagnosis, ICU, and mechanical ventilation (9, 20, and 14 days vs 7, 17, and 11days, respectively) was higher in younger patients compared to older patients.

#### Laboratory findings

Laboratory examinations for all COVID-19 patients who were critically ill are shown in ***Table 2***. CD4^+^ T lymphocytes, CD8^+^ T lymphocytes, and NKT lymphocytes were decreased, and levels of LDH, IL-6, and CD4^+^/CD8^+^ T lymphocytes were significantly increased in all 32 patients, while there were no evident differences between younger and older patients. Compared to younger COVID-19 patients under 60 years old, platelets, DBil, IBil, LDH, BNP, CRP, PCT, and IL-10 were all significantly increased in elderly patients (median platelet level, 280 vs. 136×10^9^/L; median DBil level, 3.3 vs. 12.9 μmol/L; median IBil level, 4.8 vs. 12.1 μmol/L; median LDH level, 264 vs. 415 U/L; median BNP level, 185 vs. 918 pg/mL; median CRP level, 14.6 vs. 90.5 mg/L; median PCT level, 0.091 vs. 0.234 ng/mL; and median IL-10 level, 5.36 vs. 43.60 pg/mL) (*P*-value < 0.05). White blood cells (WBCs), neutrophils, B lymphocytes, ALT, AST, De Ritis Ratio, ALP, TBil, Blood urea nitrogen (BUN), creatinine (Cr), fibrinogen, D-dimer, hypersensitive troponin T (HTT), TNF-a, and IFN-r all showed no evident differences between younger and older patients.

**Table 2:** Laboratory finding of subjects with COVID-19 in different ages.

	Normal Range	Median(IQR)			
		All patients(n = 32)	Age <60 years old(n = 13)	Age≥60 years old(n = 19)	*P*-value
**WBC, ×10**^**9**^**/L**	3.5–9.5	7.5(5.7–12.6)	7.4(6.5–14.6)	7.6(5.0–11.2)	0.404
**Neutrophil, ×10**^**9**^**/L**	1.8–6.3	5.9(4.4–11.5)	5.9(5.4–13.2)	5.6(3.9–10.6)	0.404
**Lymphocytes, ×10**^**9**^**/L**	20–50	0.58(0.34–0.95)	0.84(0.40–1.04)	0.53(0.33–0.77)	0.287
**Platelet, ×10**^**9**^**/L**	125–350	174(122–270)	280(188–325)	136(107–185)	**<0.0001**
**CD4+T, /ul**	550.0–1440.0	344(111–531)	464(286–561)	117(72–302)	0.133
**CD8+T, /ul**	320.0–1250.0	136(35–210)	145(114–238)	40(17–203)	0.161
**CD4/CD8+**	0.71–2.78	3.10(2.02–3.86)	3.03(1.55–3.45)	3.21(2.46–3.93)	0.601
**BT, /ul**	90.0–560.0	114(72–167)	115(74–194)	90(64–168)	0.740
**NKT, /ul**	150.0–1100.0	52(11–87)	68(40–88)	12(8–83)	0.270
**ALT, U/L**	0–40	24(15–49)	16(10–66)	28(18–49)	0.223
**AST, U/L**	0–40	29(20–45)	20(15–42)	31(25–46)	0.071
**De Ritis Ratio **	NA	1.21(0.64–1.65)	1.00(0.56–1.71)	1.27(0.92–1.62)	0.677
**ALP, U/L**	35–105	79(63–94)	80(66–109)	79(59–94)	0.520
**TBil, umol/L**	0–25	22.4(8.7–34.7)	11.2(6.7–29.0)	28.2(15.8–35.6)	0.126
**DBil, umol/L**	0–10	8.8(3–17.1)	3.3(2.5–8.9) (20–45)	12.9(5.2–25.2)	**0.003**
**IBil, umol/L**	0–14	6.7(4–13.8)	4.8(3.6–6.4)	12.1(4.8–17.9)	**0.011**
**BUN, mmol/L**	2.2–8.2	5.83(3.88–8.72)	4.65(3.42–6.82)	6.82(4.30–9.48)	0.136
**Cr, umol/L**	20–115	59(44–76)	45(34–63)	64(53–81)	0.054
**Fibrinogen, ug/mL**	0–5	3.67(2.70–5.43)	3.51(2.98–5.01)	4.01(2.15–5.65)	1.000
**D-dimer, mg/L**	0–0.55	1.74(0.33–10.28)	0.49(0.31–3.53)	2.71(0.78–24.96)	0.147
**LDH, U/L**	75–245	348(242–499)	264(173–423)	415(300–550)	**0.027**
**HTT, ng/mL**	0–0.1	0.015(0.007–0.047)	0.008(0.006–0.017)	0.018(0.010–0.054)	0.157
**BNP, pg/mL**	0–526	455(140–1373)	185(100–765)	918(270–3009)	**0.045**
**CRP, mg/L**	0–5	63.8(12.4–117.0)	14.6(1.4–62.8)	90.5(36.1–149.5)	**0.003**
**PCT, ng/mL**	0–0.046	0.128(0.069–0.380)	0.091(0.059–0.117)	0.234(0.085–0.484)	**0.023**
**IL-6, pg/mL**	0.00–6.61	130.58(18.95–1787.36)	98.32(12.69–138.22)	600.10(128.71–11244.62)	0.055
**IL-10, pg/mL**	0.00–2.31	9.18(4.83–43.60)	5.36(4.17–10.74)	43.60(9.88–90.86)	**0.011**
**TNF-a, pg/mL**	0.00–33.27	23.20(9.71–62.16)	23.20(9.71–62.16)	16.18(9.71–66.30)	0.931
**IFN-r, pg/mL**	38.51	9.95(2.94–20.46)	11.53(2.94–26.11)	7.46(2.94–16.55)	0.537

**Abbreviations:** WBC, White blood cell; BUN, Blood urea nitrogen; Cr, Creatinine; HTT, Hypersensitive troponin

**Fig 1:**
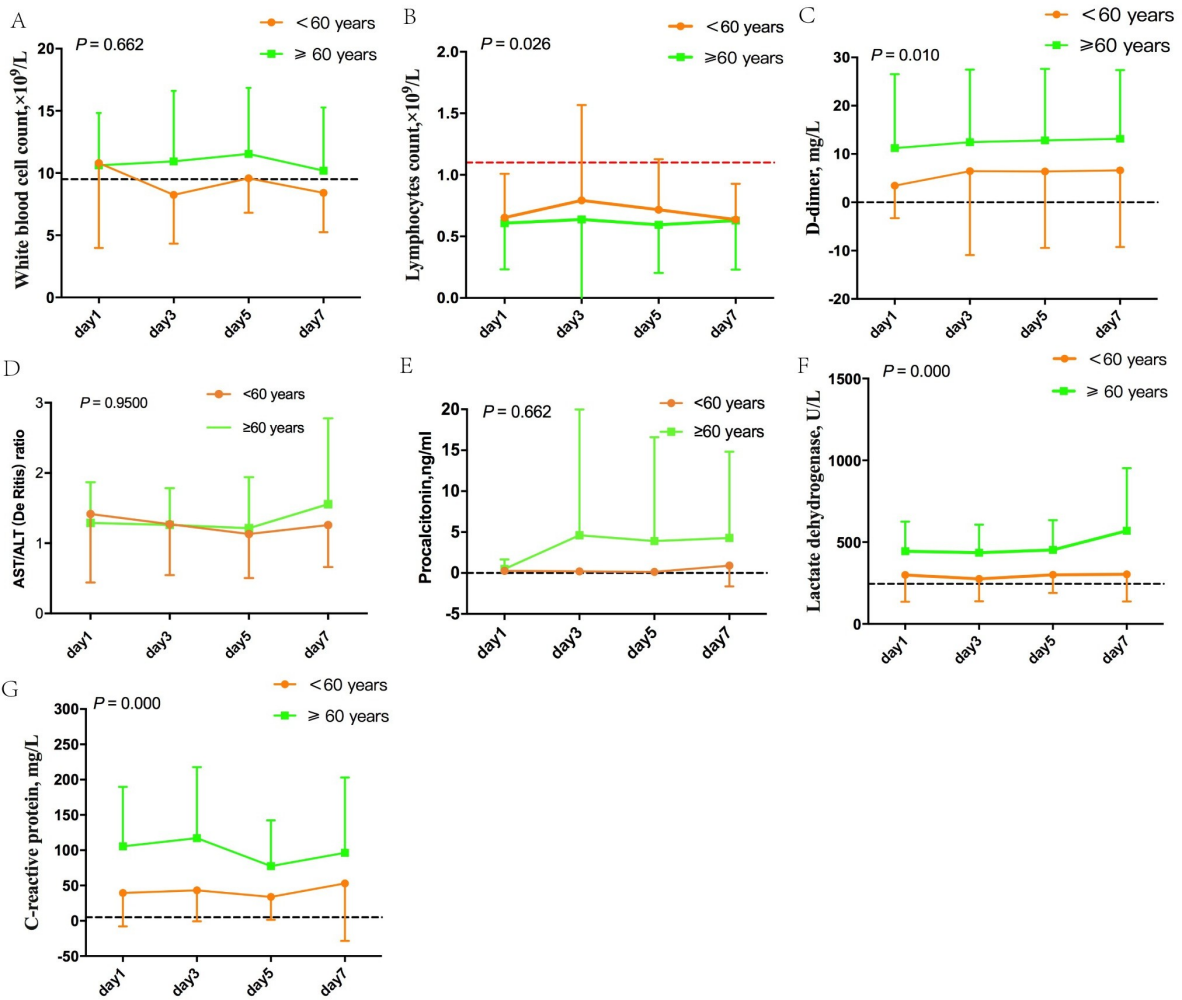
Comparison of dynamic trajectories of six laboratory parameters in COVID-19 patients between two age groups. Dynamic trajectories of six laboratory parameters were tracked on Days 1, 3, 5 and 7. Significant differences in lymphocyte counts (*P*-value = 0.026), D-dimer (*P*-value = 0.010), lactate dehydrogenase (*P*-values = 0.000) and CRP (*P*-values = 0.000) are shown in panels B, C, F and G). There were no significant differences in levels of white blood cells (*P*-value = 0.026), the De Ritis ratio (P-value = 0.950) and procalcitonin (*P-*value = 0.026), which are shown in panels A, D and E. The dash lines in black show the upper normal limit of each parameter, and the dash line in red shows the lower normal limit of each parameter.

As described in Fig 1, the dynamic trajectories of six laboratory parameters were tracked on Day 1, 3, 5 and 7. Significant differences in lymphocyte counts, D-dimer, CRP and lactate dehydrogenase were found between younger and older patients with COVID-19 (all *P*-values <0.05). There were no significant differences in levels of white blood cells (P-value = 0.026), the De Ritis ratio (P-value = 0.950) and procalcitonin (P-value = 0.026).

#### Severity of illness scores, blood gas and vital signs

Disease severity evaluation, blood gas and vital signs in younger and older patients are shown in ***Table* 3**. Compared to the younger patients under 60 years old, older subjects had significantly higher disease severity on the day of hospital admission (all *P*-value < 0.05 for CURB-65 and APACHE II). For blood gas, pH values were significantly higher in older patients (*P*-value < 0.05), and levels of PaO_2_:FiO_2_ and PaO_2_ were lower in the older patients (all *P*-value < 0.05). Lactate levels were high in both younger and older groups, but there was no evident difference between the groups. No obvious abnormal vital signs were found in any of the patients.

**Table 3:** Severity of illness scores, blood gas and vital signs of subjects with COVID-19 in different ages.

	Normal range	Median(IQR)			
		All patients(n = 56)	Age <60 years old(n = 34)	Age≥60 years old(n = 34)	*P*- value
**Severity of illness scores**					
CURB-65	NA	2(0–2)	0(0–1)	2(1–3)	**0.001**
APACHE II	NA	11(6–17)	6(5–11)	19(12–29)	**0.036**
**Blood gas**					
PH	7.35–7.45	7.459(7.423–7.503)	7.439(7.396–7.489)	7.492(7.457–7.514)	**0.031**
Lactate, mmol/L	0.5–1.7	1.8(1.6–3.1)	1.8(1.7–3.3)	2.0(1.5–3.0)	1.000
PaO_2_:FiO_2_, mm Hg	400–500	116.07(68.46–206.10)	181.95(142.32–241.93)	92.46(57.90–152.70)	**0.007**
PaO_2_, mm Hg	95–100	67.9(56.9–90.5)	76.3(64.1–103.6)	61.0(50.4–79.7)	**0.049**
PaCO_2_, mm Hg	35–45	36.2(32.7–41.2)	36.6(32.4–50.1)	36.2(33.2–38.8)	0.734
**Vital signs**					
Temperature [°C]	36.0–37.0	36.8(36.5–37.0)	36.6(36.5–36.8)	37.0(36.7–37.8)	**0.018**
Breathe[times/min]	12–20	20(18–26)	20(17–30)	20(18–25)	1.000
Sphygmus[times/min]	60–100	85(80–95)	82(78–92)	86(80–100)	0.767
MAP, mmHg	75–105	98(89–113)	100(96–118)	93(86–109)	0.084

**Abbreviations:** CURB-65, (confusion, urea, respiratory, rate, blood pressure plus age ≥65 years); (APACHE II), Acute Physiology and Chronic Health Evaluation II

#### Complications and treatment

According to the severity of COVID-19, we further analyzed complications and treatment in the different age groups. As presented in ***Table* 4**, common complications, including acute respiratory distress syndrome (ARDS) and septic shock, were more likely to be observed in the older patients (all *P*-value<0.05). Common complications observed in severe patients included DIC, arrhythmia, cardiac injury and gastrointestinal hemorrhage, and there were no evident differences between the younger and older groups.

**Table 4:** Complications and treatment of subjects with COVID-19 in different ages.

	All patients(n = 56)	NO. (%)		
		Age <60 years old(n = 34)	Age≥60 years old(n = 34)	*P*- value
**Complications**				
ARDS	20(62.5%)	5(38.5%)	15(78.9%)	**0.030**
Sepsis shock	**7**(21.9%)	0(0.0%)	7(36.8%)	**0.025**
DIC	9(28.1%)	2(15.4%)	7(36.8%)	0.178
Arrhythmia	14(43.8%)	4(30.8%)	10(52.6%)	0.289
Cardiac injury	5(15.6%)	1(7.7%)	4(21.1%)	0.625
Gastrointestinal hemorrhage	2(6.3%)	0(0.0%)	2(6.3%)	0.502
**Treatment**				
Antibacterial agents	31(96.9%)	13(100%)	18(94.7%)	1.000
Glucocorticoids	21(65.6%)	7(53.8%)	14(73.7%)	0.283
Immunoglobulin	16(50.0%)	3(23.1%)	13(68.4%)	**0.029**
Renal replacement therapy	13(40.6%)	3(23.1%)	10(52.6%)	0.147
ECMO	12(37.5%)	3(23.1%)	9(47.4%)	0.267
**Mechanical ventilation**				
Invasive	16(50.0%)	3(23.1%)	13(68.4%)	**0.029**

**Abbreviations:** ARDS, Acute respiratory distress syndrome; DIC, Disseminated intravascular coagulation ECMO; Extracorporeal membrane oxygenation.

All critically ill patients were given antiviral therapy and the antiviral treatment included Arbidol, Lopinavir/ritonavir, Oseltamivir, Interferon alpha inhalation and Ribavirin. Thirty-one (96.9%) severe patients received antibacterial agent treatment. In addition, 21 (65.6%) critically ill patients received glucocorticoid therapy, 13 (40.6%) received renal replacement therapy, and 12 (37.5%) received ECMO therapy. Patients who were 60 years and older were the most likely to receive immunoglobulin therapy (*P*-value = 0.029) and invasive mechanical ventilation therapy (*P*-value = 0.029).

## Discussion

In this study, we report 32 patients with laboratory-confirmed SARS-CoV-2 infection and assigned 60 years old as the threshold, separating the two groups by age. Both clinical and epidemiological characteristics of COVID-19 patients have been reported [8–10], but few studies have descriptively evaluated the differences in baseline clinical features, laboratory findings, severity of illness scores, blood gas and vital signs, complications and treatment of critical COVID-19 patients in different age groups. The present study first systematically evaluated the impact of age. Our findings may highlight the clinical significance in the enhanced attention towards COVID-19 elderly patients.

Notably, critical illness mortality rates were high, and the elderly patients with comorbid conditions were more likely to progress to severe illness, we analyzed the clinical characteristics and laboratory findings can help to better inform the efforts to treat COVID-19 patients in the older group. Previous research findings have mentioned that older patients might develop ARDS and tend to die after infection [4, 5, 7]. In this study, we revealed that elderly patients were also more likely to develop ARDS and septic shock. This finding might further explain the significant association of age with poor clinical outcomes observed in the previous study [11]. So, early diagnosis, careful nursing, observation and systemic treatment are of great importance for the elderly patients of COVID-19.

To better understand the influence of age on COVID-19, we first investigated the clinical and immune characteristics in COVID-19 patients in two different age groups. Results revealed that the most common presentation symptoms were fever and cough, suggesting that the virus is primarily transmitted through the respiratory tract [12]. Expectoration, fatigue, headache, and diarrhea were accompanied by fever, which may be closely related to the distribution of the viral receptor ACE2 [13]. Previous studies have indicated that the incidence of comorbidities is relatively higher in elderly patients compared to younger patients and are prone to be affected by the original disease after viral infection [14]. Our study found that the proportion of elderly patients with COPD and cardiovascular disease was higher than in younger patients. Older age was considered an independent risk factor for COVID-19 patient death [15], which is consistent with our results.

In this study, the number of lymphocytes was significantly reduced, and this decrease was more pronounced in older adults. Specifically, CD4+T lymphocytes, CD8+T lymphocytes, and NKT lymphocytes were all reduced in all critically ill patients, indicating that COVID-19 leads to impairment of lymphocytes in the immune system. Lymphocyte deficiency or incapacity in COVID-19 patients could promote disease progression, and functional exhaustion of antiviral lymphocytes was observed in COVID-19 patients [16, 17]. High-dimensional single-1 cell analysis also reveals that revealed CD4+ T and CD8+T cell depletion [18]. Human T cells are the main immune cells against viral infection and play key roles in viral clearance in the host defense against acute respiratory infections. NK cell is an important part of lymphocytes play an indispensable role in innate immunity and can contribute to the activation and orientation of adaptive immune response [19]. Our data showed that the count and percentage of NK cells obviously significantly decreased in severe COVID-19 patients. The result is similar to those of Li et a. [20] Nevertheless, B cells exhibited a relatively opposite phenomenon distinct from other lymphocytes, being within normal range in this study. There was no significant difference between the younger and older patients, which revealed that B cells were only slightly impaired in critically ill patients. Most hospitals are try to use convalescent plasma as a source of therapeutic polyclonal antibodies for treatment and investigate the efficacy of passive antibody therapies for COVID-19 and many reports suggest this method of treatment is a positive impact on clinical status. But there hasn’t been a lot of research to confirm the result [21, 22]. We can try to treat severe patients with convalescent plasma from mild patients and further explore its application value.

In addition, our study revealed that levels of AST, ALP, De Ritis ratio, TBil, DBil, IBil, BUN, Cr, fibrinogen and HTT exhibited no exceptions. Our data show that critically ill patients rarely had underlying liver injury, and this finding is similar to those of Wu et al [23]. The De Ritis ratio was first defined by Fernando De Ritis in 1957 [24]. As previously reported, the De Ritis was recognised as the ratio between AST and ALT levels (AST/ALT) in the blood serum as a good indicator of liver damage. Studies revealed that rates of De Ritis < 1.0 indicated moderate to severe liver damage, and rates higher than 1.0 indicated severe liver diseases [25]. In Table 2, compared to younger COVID-19 patients under 60 years old, the De Ritis significantly increased in elderly patients. This result suggested that the older group had a greater degree of liver function impairment. Clinically, we found that many patients exhibited arrhythmias and increased levels of BNP, while we did not observe reduced HTT. Compared to younger patients who were under 60 years old, levels of BNP were significantly increased in elderly patients. The above results may have appeared in older patients due to the basic disease. We and Zhong et al. [8] indicated that the proportion of hypertension and cardiovascular disease was higher in elderly patients compared to younger patients. Finally, we found that cytokine levels were acutely increased in severe and critical COVID-19 patients, particularly IL-6, IL-10, CRP and PCT. This result was consistent with that of Conti et al. who indicated that COVID-19 infects the upper and lower respiratory tract and further causes ARDS with consequent release of pro-inflammatory cytokines, including IL-1β and IL-6. Alimuddin Z et al. found that COVID-19 was closely associated with a cytokine storms, leading to multiple organ dysfunction [26]. Compared to younger patients under 60 years old with COVID-19, levels of CRP, PCT, and IL-10 were all significantly increased in elderly patients. Generally, patients with normal aging experience a decline in physiological immune function, and the phenomenon of immunosuppression is prone to occur in older patients, making it difficult for them to control pro-inflammatory responses [27].

As COVID-19 is an emerging disease, there is no effective therapy for it, and mechanical ventilation is the primary supportive treatment for critically ill patients. The PaO_2_/FiO_2_ ratio was lower in elderly patients than in younger patients in our study, indicating that this ratio is associated with the severity of illness and prognosis. The proportion of elderly patients administered immunoglobulins were higher than those in the younger patients, possibly due to elderly patients having low immune function.

The present study has some limitations. First, this was a single-center study involving a small number of cases, which might cause bias and limit the reliability of our result. Second, some clinical and laboratory data from the ICU were missing and couldn’t be included, such as laboratory data for days 14 and 28. Furthermore, the study data did not permit a preliminary assessment of patients at admission.

## Conclusions

In the current study, based on results from Zhengzhou, China, significant changes in severity between younger and elderly COVID-19 patients is reported. These results may be useful for clinicians to assess the risk stratification of critically ill patients. Compared to younger patients, elderly patients exhibited more severe clinical manifestations and higher mortality. Therefore, closer monitoring and effective therapeutics may be needed for elderly patients.
